# Modulation of EMG-EMG Coherence in a Choice Stepping Task

**DOI:** 10.3389/fnhum.2018.00050

**Published:** 2018-02-13

**Authors:** Ippei Nojima, Tatsunori Watanabe, Kotaro Saito, Shigeo Tanabe, Hoshinori Kanazawa

**Affiliations:** ^1^Department of Physical Therapy, Nagoya University Graduate School of Medicine, Nagoya, Japan; ^2^Japan Society for the Promotion of Science, Tokyo, Japan; ^3^Faculty of Rehabilitation, School of Health Sciences, Fujita Health University, Toyoake, Japan; ^4^Graduate School of Information Science and Technology, The University of Tokyo, Tokyo, Japan

**Keywords:** EMG-EMG coherence, anticipatory postural adjustment, elderly, risk of fall, corticospinal excitability

## Abstract

The voluntary step execution task is a popular measure for identifying fall risks among elderly individuals in the community setting because most falls have been reported to occur during movement. However, the neurophysiological functions during this movement are not entirely understood. Here, we used electromyography (EMG) to explore the relationship between EMG-EMG coherence, which reflects common oscillatory drive to motoneurons, and motor performance associated with stepping tasks: simple reaction time (SRT) and choice reaction time (CRT) tasks. Ten healthy elderly adults participated in the study. Participants took a single step forward in response to a visual imperative stimulus. EMG-EMG coherence was analyzed for 1000 ms before the presentation of the stimulus (stationary standing position) from proximal and distal tibialis anterior (TA) and soleus (SOL) muscles. The main result showed that all paired EMG-EMG coherences in the alpha and beta frequency bands were greater in the SRT than the CRT task. This finding suggests that the common oscillatory drive to the motoneurons during the SRT task occurred prior to taking a step, whereas the lower value of corticospinal activity during the CRT task prior to taking a step may indicate an involvement of inhibitory activity, which is consistent with observations from our previous study (Watanabe et al., 2016). Furthermore, the beta band coherence in intramuscular TA tended to positively correlate with the number of performance errors that are associated with fall risks in the CRT task, suggesting that a reduction in the inhibitory activity may result in a decrease of stepping performance. These findings could advance the understanding of the neurophysiological features of postural adjustments in elderly individuals.

## Introduction

Falling is a concerning health problem for the elderly population because normal aging is responsible for declines in muscular, sensory and neural control systems (Lord and Fitzpatrick, 2001; Lajoie and Gallagher, 2004). One of the risk factors identified to be related to falls in the elderly is poor reactive and volitional stepping from a standing position (Lord and Fitzpatrick, 2001; St George et al., 2007; Pijnappels et al., 2010). Previous studies have shown that a delayed execution time can be a reliable and valid predictor of future falls (Schoene et al., 2014; Delbaere et al., 2016). An anticipatory postural adjustment (APA), constituting a general form of postural accompaniment, acts to stabilize posture and equilibrium before the initiation of a voluntary movement (Massion, 1992). In the transition from stationary standing to stepping forward, the APA produces consecutive muscle activities that generate a force required to move the center of pressure (COP) backward and toward the swing leg, which is followed by a COP displacement toward the stance leg. This consecutive series of postural movements unloads the swing leg, and thus is essential for forward progression (Burleigh et al., 1994; Elble et al., 1994). Furthermore, APA has been reported to be modulated by aging and several pathological conditions (Mancini et al., 2009; Kanekar and Aruin, 2014), signifying their sensitivity and importance in the control of stepping movements.

Recent studies have indicated that the initial incorrect weight transfer, defined as APA errors, could occur when there is uncertainty regarding the direction of step initiation (Cohen et al., 2011; Sparto et al., 2013). APA is supposed to account for delayed step execution because incorrect weight shift has to be corrected prior to step initiation (Cohen et al., 2011; Watanabe et al., 2015). Given that poor executive function is a key contributor to APA errors and delayed step execution, it is essential to uncover how the cortex is related to this phenomenon, such as the involvement of corticospinal activity.

Descending neural drive from sensorimotor cortex (SMC) during movement have been extensively investigated, mostly through analysis of the frequency domain of the coupling between brain activity and muscular activity (Hansen et al., 2002; Halliday et al., 2003; Norton and Gorassini, 2006; Nielsen et al., 2008; Petersen et al., 2012; Willerslev-Olsen et al., 2015). Especially, corticomuscular coherence has been reported in the beta and gamma bands that are strongly related to corticospinal drive (Conway et al., 1995; Mima and Hallett, 1999a,b; Grosse et al., 2002). Moreover, coherence analysis has been performed on muscle pairs and shown to reflect cortical, subcortical and spinal mechanisms (Brown et al., 1999; Grosse et al., 2003). In electromyography (EMG)-EMG coherences, it was reported that during static muscle contraction patients with central nervous system (CNS) disorders, such as stroke and spinal cord injury, showed low coherence values (Farmer et al., 1993; Fisher et al., 2012). Furthermore, the beta band in intermuscular coherence can detect cortical excitability changes following transcranial direct current stimulation over the SMC (Power et al., 2006). These findings suggest that EMG-EMG coherence may reveal the presence of shared neural presynaptic input from the higher CNS and particularly from the motor cortex (Bo Nielsen, 2002), and this analysis might be a promising biomarker of corticospinal control during movements.

In studies using transcranial magnetic stimulation, corticospinal activity during quiet standing has been a focus, and its involvement in the control of posture has been suggested (Petersen et al., 2009; Kantak et al., 2013). However, corticospinal activity associated with postural preparation in a stepping task that is commonly used as an assessment for risk of falling is as yet unclear. Assessments of postural responses are generally executed in two types of stepping task: a simple reaction time (SRT) task and choice reaction time (CRT) task. In the SRT task, subjects are told which foot to response with in advance and thus can be fully prepared before an imperative stimulus. In the CRT task, some studies found that the response may be prepared in advance (Oude Nijhuis et al., 2007; Maslovat et al., 2011; Delval et al., 2012), while others did not (Carlsen et al., 2004, 2008). Conversely, we reported that the APA was prepared in advance in stepping tasks involving choice responses, and that preparatory activity could be modulated by inhibitory activity (Watanabe et al., 2016). Thus, assessing corticospinal excitability in the preparation period during stepping tasks may provide evidence for that debate, and clarifying the neurophysiological mechanisms in the stepping tasks could allow us to better understand the neuromuscular coordination of postural control.

In the present study, using EMG-EMG coherence analysis, we explored whether corticospinal activity during preparation for forward stepping would be different between the SRT and CRT tasks and how its activity contributes to APA performance. EMGs were recorded from the proximal and distal tibialis anterior (TA) and soleus (SOL) muscles. As inhibitory interneuron networks could be associated with the activation of corticospinal loop (Matsuya et al., 2017), we hypothesized that EMG-EMG coherence in the CRT task would be smaller than the SRT task because prepared APA would have to be inhibited until the proper time to start the stepping movement (Watanabe et al., 2016). Furthermore, EMG-EMG coherence would be related to some APA parameters, such as reaction time (RT) and the number of APA errors.

## Materials and Methods

### Subjects

Ten healthy elderly subjects (five males and five females) from 66 to 74 years of age (mean ± SD: 69.7 ± 2.6 years) participated in this experiment. They were all right-leg dominant by self-report and recruited from the local community. The inclusion criteria for the elderly subjects were the following: (1) no history of any neurological, orthopedic, cognitive, or psychiatric problems that interfere with balance; (2) all had normal or corrected-to-normal vision. Informed consent to participate in the experimental procedures was obtained from the subjects before experiment. The study was approved by the Ethics Committee of Nagoya University.

### Experimental Design and Task

**Figure 1:**
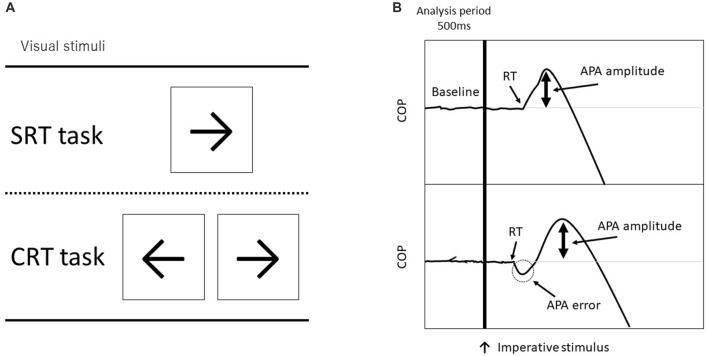
**(A)** Visual imperative stimuli used in each task (SRT, simple reaction time and CRT, choice reaction time). The CRT task consisted of a right arrow on the right side and a left arrow on the left side. **(B)** Examples of force plate data for forward step execution with the right leg. The top figure shows a non-erroneous CRT (CRT_noerr) trial, and the bottom shows an erroneous CRT (CRT_err) trial. The vertical line indicates timing of the visual imperative stimulus. The erroneous CRT trials were identified by an initial incorrect center of pressure (COP) shift toward the stance leg.

Each subject stood with their bare feet on a force plate (Tec Gihan, Kyoto, Japan). The initial standing position was the same for all participants; each foot was placed 5 cm apart from a center line on the force plate and they maintained a stationary standing position with both arms at their sides. The subjects were instructed to step forward as quickly and accurately as possible in response to a visual imperative stimulus that appeared on a computer screen just below eye level at a 1.0 m distance from the subject. Forward stepping distance was also determined to be 45% of the participant’s leg length, measured from the greater trochanter to the floor. Before each trial, they were instructed to the equal distribution of weight, which was confirmed by the position of the COP obtained online from the force plate. The visual stimulus of an arrow (← or →) appeared on either the right or left side of the fixation point on the computer screen, the participant stepped forward in response to a visual imperative stimulus (“←” was assigned to the left leg and “→” was assigned to the right leg) onto a wooden plate placed right in front of the force plate and then they brought the other leg alongside (Figure 1). After stepping, they were required to move back to the same starting position, which foot position was marked by tape, and prepare for the next trial.

The duration of each trial was 10 s. All visual stimuli were presented in black on a white-screen background. The trial started with the fixation point presented in the center of a computer screen, which was followed by a visual imperative stimulus of a black arrow (500 ms long).

Each participant performed three blocks of each task. In the SRT task, one block was comprised of 20 trials with right-pointing arrows, and the participants were required to step forward with the right leg. In the CRT task, 10 left- and 10 right-pointing arrows were randomly displayed in each block, and the participants were required to step forward with the leg that corresponded to the direction of the arrow.

### Data Recording

Recorded signals (COP and EMG signals) were analyzed off-line with custom-written software routines (MatLab R 2014a, The MathWorks, Natick, MA, USA). EMGs were recorded bilaterally in the proximal part of TA and the distal part of TA and SOL muscles using surface Ag-AgCl electrodes (interelectrode distance of 2.0 cm). The pairs of electrodes for the TA muscle were placed at least 10 cm apart from each other to minimize the potential risk of cross talk (Hansen et al., 2005). The surface electrodes were attached the belly of each muscle after cleaning with alcohol and gentle abrasion of the subject’s skin. The signals were amplified and band-pass filtered (1–1000 Hz), then digitized and sampled to a computer using a conventional EMG machine (Nihon Kohden, Tokyo, Japan). The signal acquisitions and visual stimulus generation were performed with a customized LabVIEW program (National Instruments, Austin, TX, USA).

### Data Analysis

#### APA Parameters

The COPs were calculated from the data obtained from the force plate (Figure 1). The COP signals at a sampling rate of 1000 Hz were filtered with a 50 Hz low-pass, fourth-order, and zero-lag Butterworth filter before further processing. To differentiate the APA initiation from mere oscillation of the COP, its onset was defined using the COP movement speed (Delval et al., 2012). A threshold was set as follows: a COP speed >100 mm/s for at least 3 ms. The erroneous APAs in the CRT task were identified by the mediolateral deviation of COP toward the stance leg at the time of reaction (Watanabe et al., 2016). In addition, we measured the APA amplitude, which is the peak mediolateral COP point in the APA phase. For trials with error, the APA amplitude was measured in the first peak APA. Thus, the APA amplitude during a trial with an APA error indicates the degree of erroneous APAs. Moreover, the mean mediolateral COP sway for 500 ms before imperative stimulus was calculated to assess COP displacement during the stationary stance.

#### Coherence

EMG signals were pre-processed with a high-pass filter at 40 Hz to delete noise and interference (i.e., motion artifacts and electrode cable movement artifacts). Although there has been an extensive debate on the need of rectification of the EMG signal for coherence analysis, the effect or role of rectification on the EMG-EMG coherence has not been confirmed conclusively yet. In addition, the rectified EMG has been used to examine the EMG-EMG coherence in most previous studies with the similar tasks (Boonstra et al., 2008; Obata et al., 2014). We, therefore, have applied the rectified EMG data for the following coherence analysis to compare our data with the previous studies. Spectral analysis was performed to investigate coupling of muscle pairs in the frequency domain, using the discrete Fourier transforms of the rectified EMG segments (500 data points each). The procedures for calculation of coherence between two signals have been described in a previous publication (Halliday et al., 1995). Briefly, coherence is an extension of Pearson’s correlation coefficient in the frequency domain, and coherence between signal *x* and signal *y* can be obtained by normalizing the square of the cross-spectra by the auto-spectra using the following equation:

$${\left|R_{xy}(\lambda )\right|}^{2}\;=\;\frac{{\left|f_{xy}(\lambda )\right|}^{2}}{f_{xx}(\lambda )*f_{yy}(\lambda )}$$

By an average of the periodograms across all trials, estimates of the spectra were constructed and *fxx* (λ) and *fyy* (λ) were used to represent the power spectra of processes *x* and *y*, respectively, for a given frequency λ.

A significant coherence was defined as being greater than the 95% confidence limit, which was computed using by 1-1/(0.95)^[1/(*N*−1)]^, where *N* (60 trials) represents the number of disjoint segments used for the coherence estimate. In the present study, we calculated coherences of three paired muscles: intramuscular (proximal and distal within one leg) and intermuscular (bilateral proximal TA muscles and bilateral SOL muscles, respectively) coherence, because the TA predictively works in forward stepping while the SOL is to start stepping behavior (Couillandre et al., 2002; i.e., they work for different purposes).

EMG-EMG coherence estimates provide measures of the fractions of activity in one surface EMG signal at a given frequency that could be associated by the activity in the other surface EMG signal. Its value can quantify the strengths and ranges of the frequencies of the common synaptic inputs that were distributed across the spinal motoneuron pool (Hansen et al., 2002; Petersen et al., 2010). We confirmed that high coherence across all frequency bands, which may result from cross-talk between pairs of electrodes, was not observed. We carefully recorded EMG data to avoid the influences of motion artifacts. In addition, the time frequency coherence analysis was computed using a sliding window of 250 ms with an increment of 10 ms through the entire section of interest. A total window length of 500 ms was used, corresponding to the period of EMG activity prior to the imperative stimulus.

### Statistical Analysis

Prior to data analyses of the trials, trials in which: (1) RTs were faster than 3 SD below the mean of each block (i.e., guessed response); (2) RTs were slower than 3 SD above the mean of each block (i.e., overthought response); or (3) subjects stepped with the wrong foot or failed to step were excluded.

For the APA parameters, three groups (SRT, non-erroneous CRT (CRT_noerr) and erroneous-CRT (CRT_err)) were examined by one-way measures analysis of variance (ANOVA).

For a statistical comparison of the amount of EMG-EMG coherence in each task, the coherence estimates for each paired EMG signal were normalized by hyperbolic tangent transformation. To quantitatively evaluate the magnitudes of the coherence between paired EMG signals, the amount of coherence was averaged over a range within the 8–15 Hz (alpha) and 15–30 Hz (beta) frequency bands, as in previous studies (Boonstra et al., 2008; Obata et al., 2014). In coherence parameters, the effects of task (SRT and CRT) and groups (intramuscular TA, intermuscular TA and intermuscular SOL) were assessed with a two-way repeated measures ANOVA. *Post hoc* comparisons were performed with Bonferroni’s correction when necessary. These statistical analyses were conducted using EZR, which is a modified version of R commander (version 1.6-3; Kanda, 2013). The significance threshold was set at 0.05. In addition, we investigated the relationship between the APA parameters and the coherence of all paired EMG signals using Spearman’s correlation. In the correlation analysis, because this study has small data sets, we used bootstrap techniques to estimate a model’s optimism and compute 95% confidence intervals (CI) using a 1000 bootstrap resample.

## Results

### APA Parameters

No complete step errors were observed in the present study. Every subject completed 60 trials of the SRT and CRT tasks. Thus, the total number of trials was 1200 and 24 trials (2%) were excluded. In the CRT task, the mean APA error rate (mean ± SD) was 23.8 ± 5.8% (the right step: 21.3 ± 11.5%, the left step: 26.3 ± 8.4%). The error rates of the right and left steps were not significantly different (*p* = 0.382), indicating that the dominant-leg step was not influenced on APA preparation. In the error rate, there was no significant difference between the first and last blocks (30 trials) of stepping task in the CRT task (*p* = 0.545), indicating that there were no effects of learning or fatigue on postural performance as the blocks progressed.

**Figure 2:**
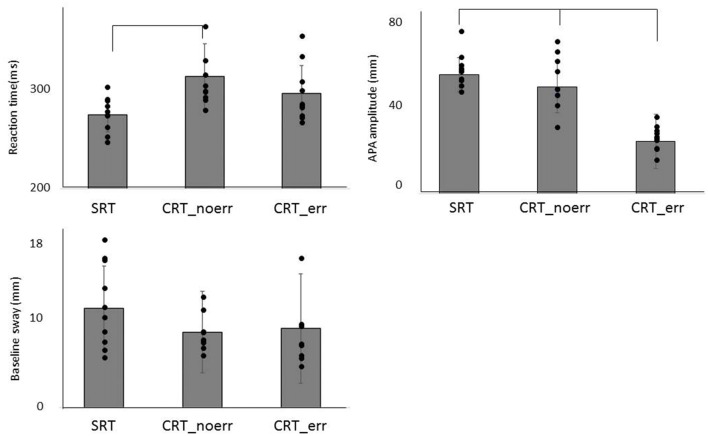
Mean reaction time (RT), anticipatory postural adjustment (APA) amplitude, and baseline sway are shown for each condition (SRT, CRT_noerr and CRT_err).

The mean values of RT and the COP displacement (APA amplitude and baseline sway) for each task are shown in Figure 2. One-way ANOVA revealed significant differences among the three groups (*F*_(2,27)_ = 5.32, *p* = 0.011). *Post hoc* analysis confirmed that RT in the SRT was significantly shorter than CRT_noerr (*p* = 0.009). Also, there was a significant difference between groups in APA amplitude (*F*_(2,27)_ = 7.43, *p* = 0.003). *Post hoc* analysis demonstrated that the amplitude for the CRT_err was smaller than that for the CRT_noerr (*p* = 0.003) or SRT (*p* = 0.041) tasks. On the other hand, there was no significant difference between groups in the mean mediolateral COP sway during the baseline period (*p* = 0.87).

### EMG-EMG Coherence

**Figure 3:**
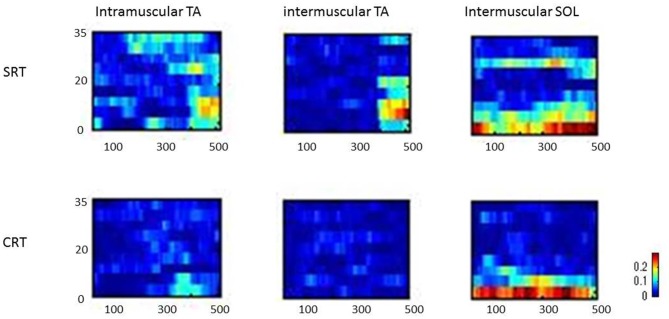
Representative time frequency analyses of coherence (intramuscular tibialis anterior (TA), intermuscular TA and intermuscular soleus (SOL)) are shown for SRT and CRT.

Figure 3 shows typical individual time frequency coherence data in both tasks for 500 ms prior to the imperative stimulus. Time frequency representations of the difference in power between the SRT task and the CRT task were clearly observed around the 10–25 Hz frequency band from 100 ms prior to imperative stimulus.

There were no subjects with high coherence across all frequency bands; thus, cross-talk between the two surface electrodes’ recordings is unlikely to explain the data observed (Hansen et al., 2005).

**Figure 4:**
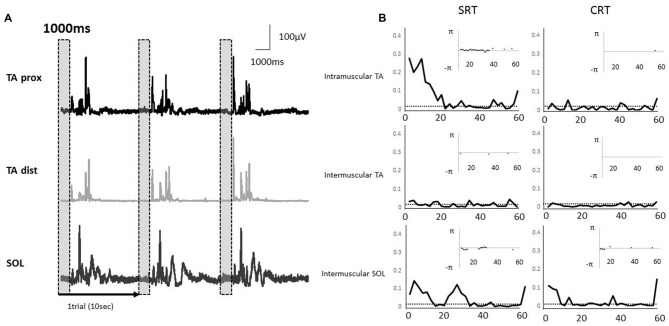
**(A)** Rectified electromyography (EMG) signals during a stepping task in a representative subject. Each EMG signal was segmented into epochs of 1000 ms that corresponded to the fixed period of paired muscle activity prior to the imperative stimulus. The EMG activities of proximal and distal TA muscle and SOL muscle were used in each coherence analysis. The vertical and shaded dashed box indicate the analysis periods. **(B)** A representative example of EMG-EMG coherence in the muscle pairs (intramuscular TA, intermuscular TA and intermuscular SOL). The horizontal dashed lines indicate the 95% confidence limit of coherence. The calculation of phase spectrum was implemented as the argument of the cross-spectrum for the estimation of the timing relationships between the EMG signals, and this figure showed the subplot that is inserted in the coherence plot. The phase spectrum is defined over the range, and the y axis is showed in radians. The phase spectrum is valid in the frequency band in which the coherence shows significantly increase, and hence, only those regions are illustrated in the phase plots.

Typical examples of rectified surface EMGs in the SRT task and the coherence functions with the phase functions for each task were shown for intramuscular TA, intermuscular TA and intermuscular SOL in Figure 4. EMG-EMG coherences were seen in the alpha and beta bands of intramuscular TA and intermuscular SOL in the SRT task, whereas there was very little coherence between each muscle pair in the CRT task (Figure 4).

**Figure 5:**
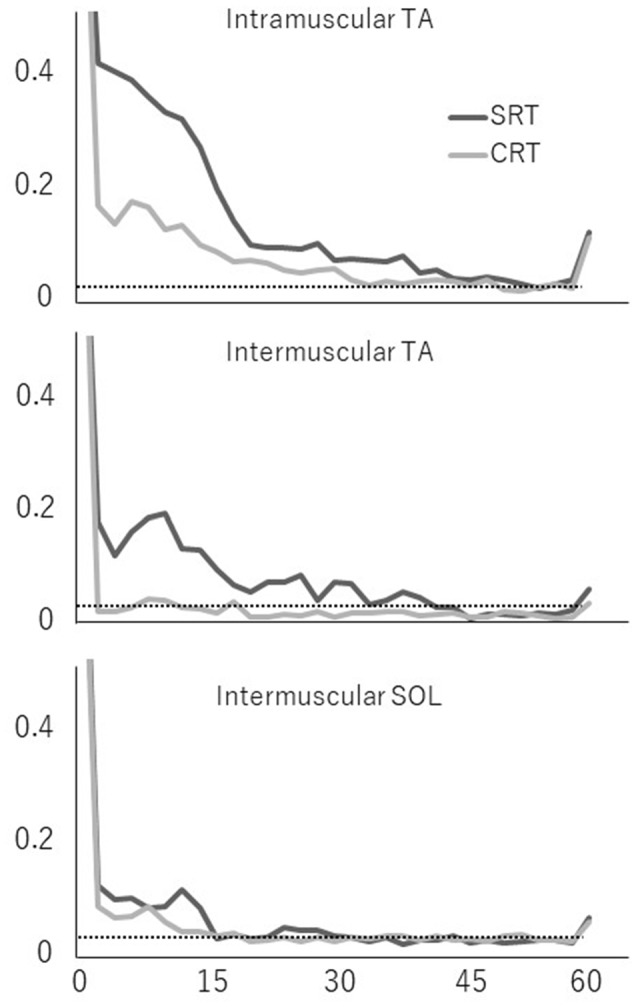
Mean coherence of each paired EMG signal (intramuscular TA, intermuscular TA and intermuscular SOL) in each task (SRT and CRT). The 95% confidence level is presented with horizontal dashed lines.

**Figure 6:**
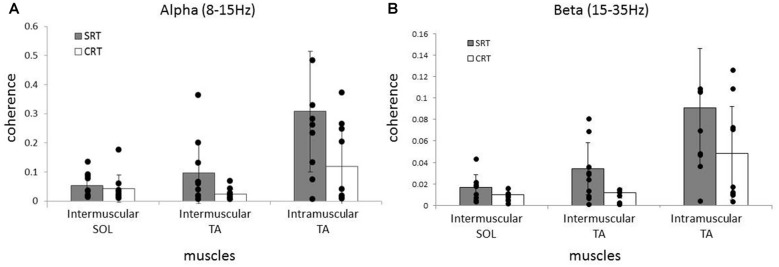
Comparison of average alpha **(A)** and beta **(B)** coherence across three paired muscles (intramuscular TA, intermuscular TA and intermuscular SOL) in each task (SRT and CRT). For the alpha band, the average coherence of all paired muscles in the SRT task was significantly higher than the CRT task. For the beta band, there were also significant differences in the average coherence for all paired muscles in the SRT task compared with the CRT task.

Averaged coherence spectra of all paired EMGs are shown, to describe the features for each task (Figure 5). The averaged coherence in the SRT task appeared greater around the alpha band than in the CRT task. More specifically, the averaged coherence around the beta band of intramuscular TA and intermuscular TA in the SRT task seemed to be greater than the CRT task. The mean coherence values for each frequency band are shown separately for each task (Figure 6). In the alpha band, a two-way ANOVA revealed a significant main effect of task condition (*F*_(2,54)_ = 8.88, *p* = 0.004) and muscles pairs (*F*_(2,54)_ = 12.26, *p* < 0.001), but there was no significant interaction (*F*_(2,54)_ = 2.89, *p* = 0.064). *Post hoc* analysis also showed that there was no significant difference in intramuscular TA (*p* = 0.132), the intermuscular TA (*p* = 0.184) and SOL (*P* = 0.884). In the beta band, a two-way ANOVA also found a significant main effect of task condition (*F*_(2,54)_ = 7.97, *p* = 0.007) and muscles pairs (*F*_(2,54)_ = 16.93, *p* < 0.001), but there was no significant interaction (*F*_(2,54)_ = 1.48, *p* = 0.238). *Post hoc* analysis also showed that there was no significant difference in intramuscular TA (*p* = 0.259), the intermuscular TA (*p* = 0.083) and intermuscular SOL (*P* = 0.310). These results indicate that EMG-EMG coherence in both frequency bands is greater in the SRT task than the CRT task.

**Figure 7:**
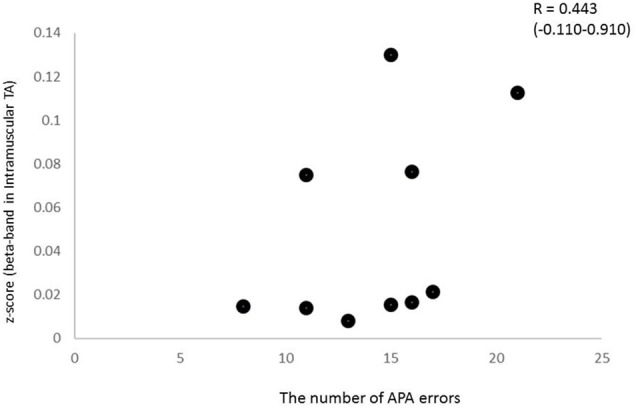
Relationship between beta intramuscular TA coherence and the number of APA errors (Spearman’s *R*^2^ = 0.443, *p* > 0.1), although a trend was present.

To characterize the relationship between beta band coherence (intramuscular TA in the CRT task) and performance (the number of APA errors), we calculated the non-parametric Spearman’s correlation coefficient. In the present study, the 95% CI calculated using the bootstrap method showed large intervals for some coefficients. In addition, relationship between beta coherence and the number of erroneous APAs in the CRT task showed no significant correlation, although a trend was present (Spearman’s rho = 0.443, *p* = 0.2; Figure 7). There was no clear relationship between other APA parameters and EMG-EMG coherence.

## Discussion

We have demonstrated in the current study that the intramuscular and intermuscular EMG-EMG coherences of lower leg in the alpha and beta bands in the CRT task were less than the SRT task. We argue that this finding could reflect inhibitory mechanisms influencing corticospinal activity during the preparation periods of stepping tasks requiring choice responses. In addition, the intramuscular TA coherence in the beta band tended to correlate positively with the number of APA errors, suggesting that the occurrence of postural errors in the CRT task could be related to a reduced ability to modulate corticospinal activity until the appropriate time to start stepping movement.

### Coherence Analysis

In the present study, we found a higher EMG-EMG coherence in the SRT task than the CRT task when preparing for forward stepping. Subjects were instructed to keep standing quietly, and we confirmed that there were no differences in COP movements between the SRT and CRT tasks during the baseline period. Previous studies reported that the RT in the stepping task was dramatically reduced by a loud auditory stimulus (LAS), suggesting advanced preparation of APA when the required stepping leg is known in advance (MacKinnon et al., 2007; Delval et al., 2012). The underlying neural mechanisms of the findings were that the LAS triggered the APA prepared and stored in the subcortical region. A greater EMG-EMG coherence during the SRT task in the alpha band, which was proposed to originate from subcortical circuits (Grosse et al., 2002; Hansen et al., 2002), suggests that increased subcortical activity is associated with APAs.

However, our previous study regarding APA preparation during the CRT task using the LAS reported that APAs were prepared in advance as in SRT tasks and the APA was suggested to be inhibited from release until the proper time to initiate a step (Watanabe et al., 2016). The findings demonstrated that neural activation for APA preparation and neural inhibition for withholding APA release operate simultaneously. Furthermore, cortical modulation during postural control was reported in many previous studies (Jacobs and Horak, 2007; Jacobs et al., 2009; Yakovenko and Drew, 2009; Chang et al., 2010; Tard et al., 2013). From these findings, we propose that the subcortical activities responsible for the generation of APAs may need to be modulated by cortical activities during a stepping task involving choice responses to initiate an APA appropriately, and this might have been reflected by the smaller coherence in the alpha band during the CRT task.

Oscillatory activity in the beta band in the SMC plays a crucial role in motor control (Van Wijk et al., 2012), and studies on EMG-EMG coherence have suggested that activation within the beta band reflects the oscillatory activity in the corticospinal pathway that originates from the SMC (Mima and Hallett, 1999a; Grosse et al., 2002; Fisher et al., 2012). During gait, the intramuscular TA coherence in the beta to gamma bands has been reported to exist at swing phase, and improving the ankle control parallels maturation of the corticospinal track (Petersen et al., 2010), suggesting that intramuscular TA coherence might be reflected in corticospinal activity from the cortex. In the present study, intramuscular TA coherence in the beta band was smaller in the CRT than the SRT task. Therefore, in addition to the signals from the subcortical areas to generate the APAs, there might have been signals from the cortex, and the reduced coherence in the CRT task might reflect inhibitory signals from other cortical areas.

In bilateral EMG activity (intermuscular TA and intermuscular SOL), previous studies suggested the involvement of subcortical circuits (Grosse et al., 2003; Boonstra et al., 2008) because alpha oscillation of EMGs from bilateral muscles are generally not synchronized with cortical activity (Baker and Baker, 2003). The origin of alpha oscillations in bilateral synchronization appears to be multifactorial in origin, and it is an issue of ongoing debate. Furthermore, bilateral synchronization around the beta band has been proposed to originate from descending projections from non-cortical areas, such as the reticulospinal system, or networks within the spinal cord (Boonstra et al., 2009). Moreover, a recent study has reported that bilateral synchronization increases during bimanual coordination task (de Vries et al., 2016). From these findings, the smaller coherence during the CRT task in the present study might be attributed to the modulation of subcortical circuits by inhibitory signals from cortical areas. However, since signals from cortical and subcortical areas cannot be separated clearly in the present study, further investigation on this issue will be necessary.

In addition to these findings, the representative time frequency coherence also allowed us to assess the coupling between the two signals over different time scales. The frequency ranges of interest were chosen in accordance with the results obtained by Fourier cross-spectral analysis. Typical examples of both tasks clearly showed that corticospinal activity occurred from 100 ms prior to the imperative stimulus, indicating the preparation for the forward step in the SRT task.

During quiet standing, a continuous ankle plantarflexion and dorsiflexion torque is needed to stabilize the ankle joint. Because the center of mass is located in front of the ankle joint, however, the plantarflexors are mainly active to stabilize the body, and the dorsiflexors are rarely activated (Masani et al., 2008). In the present study, the participants were not merely standing but preparing to initiate a step movement. This might have caused subjects to unconsciously increase TA muscle activity, which allowed us to investigate the coherence within and between the TA muscles.

Regarding the time frequency coherence analysis, varieties of methods have been used to investigate time-varying properties of non-stationary neuronal signal (Zhan et al., 2006). Bruns (2004) has reported that Fourier transform and wavelet transform approaches are mathematically equivalent spectral analysis approaches although wavelet-based coherence is also applicable to non-stationary signal and have become prevalent in some neuroscience community for time-frequency analysis (Lachaux et al., 2002). Allen and MacKinnon (2010) have further suggested that wavelet transform suffers from trade-off between temporal and spectral resolution similar to Fourier transform.

### APA Parameters

In regard to APA parameters, the RT was shorter in the SRT task compared to the CRT task without errors, and the APA amplitude in the CRT task with errors was smaller than the other two conditions. The faster RT in the SRT task is reasonable and consistent with many previous studies, and this result indicates that preparation for forward stepping in the SRT task is completed in advance. By contrast, there was no significant difference in the RT between the SRT task and the CRT task with errors. This implies that the improper release of APAs before the appropriate timing is related to the occurrence of APA errors. Furthermore, increased APA errors in the elderly compared with younger individuals have been reported (Cohen et al., 2011; Sparto et al., 2013), suggesting that APA errors are also related to executive function, especially inhibitory control.

There was a trend toward association between intramuscular TA coherence in the CRT task and the number of APA errors, although it was not significant. Since the intramuscular TA coherence in the beta band might reflect cortical activity, this relationship suggested that this measurement might become a clinical neurophysiological biomarker for physical function in elderly individuals. Conversely, some previous studies have suggested a negative association between beta band corticomuscular coherence and fine motor performance within young adults (Kristeva et al., 2007; Witte et al., 2007; Graziadio et al., 2010; Johnson and Shinohara, 2012). However, these studies employed a unilateral task or a relatively simple task, and there has been no study investigating the corticospinal activity associated with postural control tasks. This finding supports the idea that the smaller EMG-EMG coherence recorded during a choice stepping reaction could reflect cortical activity, including the inhibitory function, which may be related the APA errors. Because its relationship with clinical outcomes such as a balance scale is unclear in the present study, however, further investigations need to be conducted to clarify the clinical significance.

### Limitation

We realize that this study has several important limitations, some of which are quite prominent. First, the analysis window (1000 ms) was not enough for analysis in the low frequency band. Regarding EMG-EMG coherence of the low frequency band, it has been proposed to be associated with postural sway (Danna-Dos-Santos et al., 2014; Obata et al., 2014; Boonstra et al., 2015). Second, we need to recognize the possibility of cross-talk between pairs of EMG recordings. Although we attempted to minimize cross-talk by placing the electrodes as distant from each other as anatomically possible, we cannot completely rule out the possibility. However, high coherence over a wide range was not observed in the analyzed coherence estimate in the present study. Thirdly, it is important to note that the small sample size implies a lack of statistical power for EMG-EMG coherence analysis to draw definitive conclusions. We implemented the bootstrap method to estimate 95% CI of correlation coefficient, but analysis may need to be replicated in large cohorts. Although our results revealed the slight trend between the strength of coherence and APA, potentially suggesting the clinical significance of the coherence analysis as an assessment of risk of falls in elderly populations, we must interpret the result very cautiously. Moreover, the effect of EMG rectification on the coherence would need to be clarified in future studies. In addition, this study was conducted in elderly individuals to investigate the neurophysiological mechanisms of the tasks associated with the risk of falling. Although aging induces considerable changes in the CNS as well as the neuromuscular systems that are related to postural balance, the effects of aging on this task might need to be investigated.

## Conclusion

Differences in corticospinal activities between the SRT and CRT tasks were identified in elderly individuals during step initiation. Specifically, we identified that they were weakened in the choice reaction task, and this finding is consistent with our previous study (Watanabe et al., 2016) demonstrating that the preparatory activity of APA was modulated by inhibitory processes when the leg with which to make a step response was not known beforehand (i.e., the CRT task). Additionally, a trend for correlation between beta coherence and the number of APA errors was observed in the CRT task. To our knowledge, the current study is the first to find changes in corticospinal activity associated with stepping reaction tasks. These findings may shed light on the mechanisms underlying stepping tasks and provide new insight into the features and strategies of postural adjustments.

## Author Contributions

IN and TW designed the study and drafted the manuscript. IN, TW and KS performed the experiment. IN, ST and HK analyzed the data. IN and HK interpreted results of the experiment. All authors approved the final version of the manuscript.

## Conflict of Interest Statement

The authors declare that the research was conducted in the absence of any commercial or financial relationships that could be construed as a potential conflict of interest.
